# Oxygen Vacancy in WO_3_ Film-based FET with Ionic Liquid Gating

**DOI:** 10.1038/s41598-017-12516-y

**Published:** 2017-09-25

**Authors:** Hossein Kalhori, Michael Coey, Ismaeil Abdolhosseini Sarsari, Kiril Borisov, Stephen Barry Porter, Gwenael Atcheson, Mehdi Ranjbar, Hadi Salamati, Plamen Stamenov

**Affiliations:** 1 School of Physics and CRANN, Trinity College, Dublin 2, Ireland; 2 Department of Physics, Isfahan University of Technology, Isfahan, 84156-83111 Iran; 3 Computational Physical Sciences Research Laboratory, School of Nano-Science, Institute for Research in Fundamental Sciences (IPM), P.O. Box, 19395-5531 Tehran, Iran

## Abstract

Ionic liquid gating is a versatile method for inducing a metal-insulator transition in field-effect transistor device structures. The mechanism of carrier doping in metal oxide films is under debate. Ionic liquid gating of a WO_3_ film-based field effect transistor is discussed in this report. Flat and relatively smooth WO_3_ films were deposited on SrTiO_3_ substrates by pulsed laser deposition. Swept and constant gate voltage characteristics are measured in both argon and oxygen atmospheres. The results show a clear dependence on the oxygen pressure of the experimental chamber. Metallic behavior in the films is attributed to oxygen vacancy formation in the WO_3_ layer induced by the high electric field at the oxide-ionic liquid interface. The density of states of a monoclinic supercell of oxygen deficient WO_3_ was studied by density functional theory (DFT). Calculated W and O partial densities of states verify metallic behavior even at dilute oxygen vacancy concentrations and show the role of W and O orbitals in the conductivity.

## Introduction

Much progress has been made to improve the performance of Field Effect Transistors (FET) in the last decade^1–4^. An interesting laboratory-scale approach is to introduce an electric double layer FET, using polymers or Ionic Liquids (ILs) as the gate dielectric^5–8^. Due to the large capacitance obtained at the interface of the IL and the transistor channel, a high carrier density (10^14^–10^15^ cm^−2^) and high on/off current ratio are achievable. These new types of FETs are also attractive as they can operate at low gate voltages of less than 2 V, unlike traditional gate dielectrics. As an example, Takenobu *et al*.^9^ made an electric double layer FET using a MoS_2_ thin film and an ion gel consisting of PS–PMMA–PS and EMIM-TFSI with a high on/off current ratio of 10^5^, Also Kubozono *et al*.^10^ recorded an on/off current ratio of ~10^7^ on an FET with a one-dimensional hydrocarbon semiconductor. Moreover, an insulator to superconductor phase transition is observed in IL-based FETs in thin films of ZrNCl^11^, SrTiO_3_^12^, MoS_2_^13^, La_2–x_Sr_x_CuO_4_^14^ and KTaO_3_^15^, where T_c_ ranges from 0.04 to 14 K. Also a paramagnetic to ferromagnetic transition is recorded in IL- FET transistors of Co-doped TiO_2_^16^and Mn-doped Bi_2_Te_3_Se^17^ films.

Recently metal oxides have been used as channels in IL-based FETs because of their unique physical properties such as transparent conductivity, light emission and energy storage capacity^18^. A metal to insulator transition (MIT) has been recorded in thin films of some of these oxides, when a gate voltage is applied to the ionic liquid electrolyte^19–21^. A colossal field effect via IL gating has been demonstrated on the strongly correlated perovskite nickelate SmNiO_3_^22–25^. Nakano *et al*.^20^ proposed that the metallic behaviour is due to the direct injection of electric carriers into the structure. In this theory, collective bulk carrier delocalization is said to occur due to the electrostatic accumulation at the interface when a gate bias is applied to the ionic liquid dielectric. Natelson *et al*.^26^ proposed that a metal-insulator transition in VO_2_ is due to the electrochemical doping of hydrogen atoms from the IL to the surface of single-crystalline nanowires. Parkin *et al*. reported that the gate bias produces a phase transition that changes the crystal structure of VO_2_^27^ and TiO_2_^19^ films. They propose that the electric field at the oxide-IL interface leads to loss of oxygen and the creation of oxygen vacancies inside the film. Zou *et al*.^28^ used XRD and Raman spectroscopy during the ionic liquid gating to map the depth of oxygen vacancies created. Their DFT calculation also supports the theory of metallization via oxygen vacancies. A dual ion (hydrogen- and oxygen-based) phase transformation is proposed by Lu *et al*. [N. Lu *et al*., Nature 546, 124 (2017)] that can be regarded as the sum of the Parkin and Natelson models. In spite of the various different theories used to explain the effect, it is generally accepted in the literature that the mechanism of metallization and carrier injection in the channel is controlled by an electrochemical surface reaction^5,27,29^. IL gating has been previously demonstrated to be a viable approach for high-speed, low-voltage control in FET-transistors^30^.

WO_3_ has recently been used as a channel in IL-gated FETs in a number of studies^31–34^. An MIT is observed in thin-film hexagonal phase WO_3_ based FETs with ionic liquid gating^32^. It has been shown that liquid gating changes the crystal structure of WO_3_ films^33^. Parkin *et al*. recorded expansion and contraction of the WO_3_ lattice with applied gate voltage, along with a high current ratio as high as 10^7^ ^31^. The formation of oxygen vacancies which was indicated in their results, is different from the collective metallization proposed by Nakano *et al*.^20^. The magnitude of the electric field is estimated to be in the order of 10 MV cm^−1^, at the oxide-electrolyte interface^35^ and it is therefore able to remove O^2−^ ions from the WO_3_ surface. The bond dissociation energy or enthalpy to remove an oxygen ion from the WO_3_ structure is 6.4 eV, whereas the corresponding energy in the VO_2_ structure is 6.7 eV^36^. It follows that the high electric field produced at oxide-IL interface should be more efficient at creating oxygen vacancies in WO_3_ films than in VO_2_ in a similar FET configuration. The energy needed to create a neutral oxygen vacancy, leaving behind two electrons that occupy states at the bottom of the conduction band or donor levels below the conduction band edge will be a few tenths on an eV^37^. Furthermore, IL-gated FETs, with WO_3_ films as the transistor channel are potential candidates for electrochromic devices^38^. It has been demonstrated that using a liquid lithium based electrolyte (0.5 M LiClO_4_ -PC) as dielectric in an FET can lead to an on/off current ratio of up to 10^6^, as well as a reversible colour change, thus making the devices suitable as an electrochromic transistor system^39^.

In this study, the IL gating effect on the surface of WO_3_ films is examined. High quality WO_3_ films are deposited on SrTiO_3_ (001) substrates by pulsed laser deposition (PLD)^40^. An ionic liquid is used as dielectric in our electric double layer FET device. IL gating at different oxygen partial pressures is implemented in order to investigate the effect of oxygen concentration on the metal-insulator transition. It is shown that oxygen vacancies play an important role in the metallization of WO_3_ films, which is also verified by our DFT calculations. The WO_3_ film color is also demonstrated to change reversibly when a voltage is applied to the IL, suggesting the possibility of using ILs as liquid electrolytes in electrochromic applications too.

## Results and Discussion

**Figure 1:**
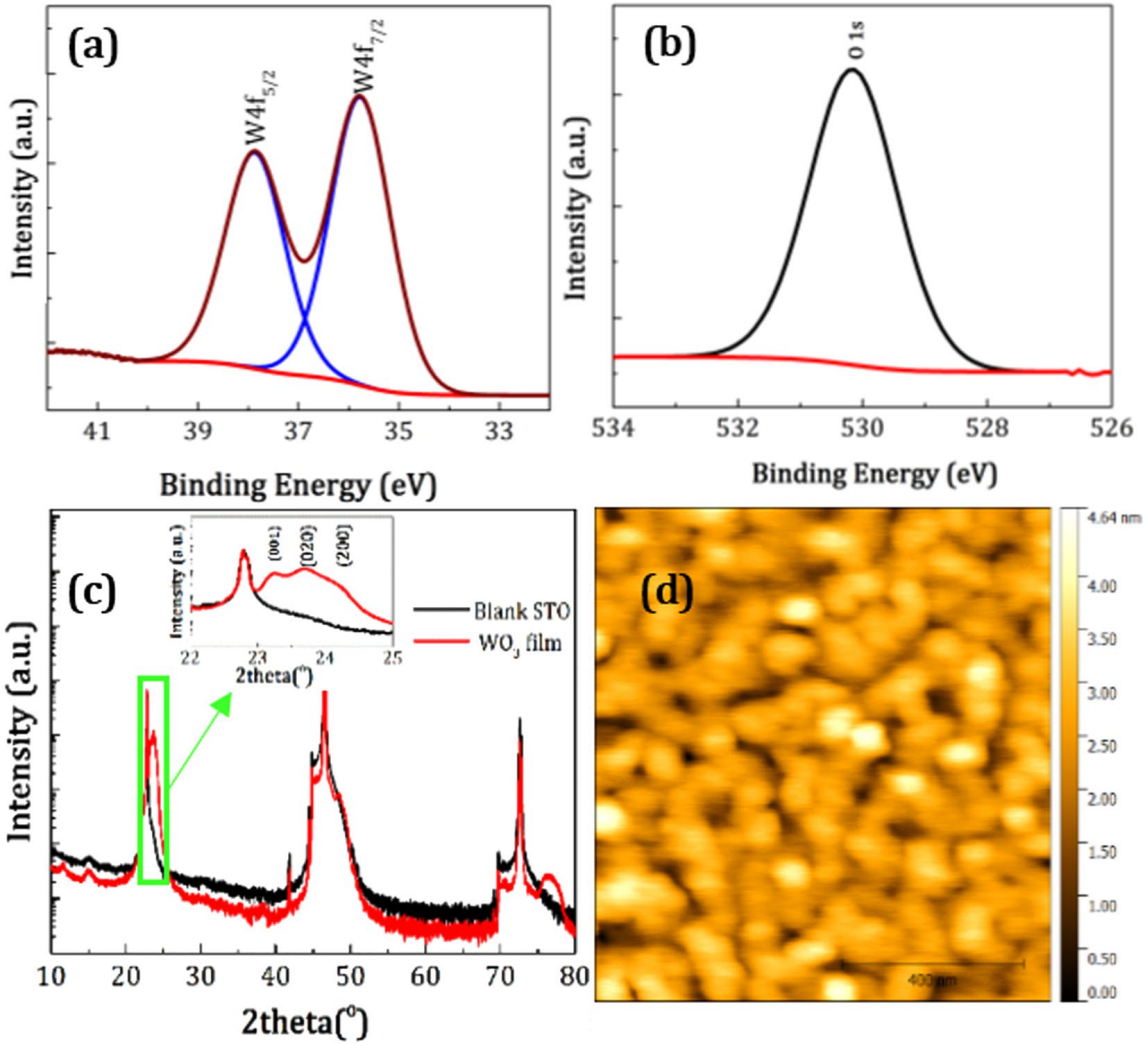
Characterization of the surface of the WO_3_ thin film on STO substrate deposited with PLD method. (**a**) Core level W 4 *f*, (**b**) O 1 *s* XPS spectra, (**c**) X-ray diffraction of WO_3_ film and STO substrate, inset shows the range of 22–25° of 2θ, and (**d**) AFM image of the WO_3_ film surface. The film thickness is 50 nm.

We investigated first the chemical and structural properties of the thin films by X-ray Photoemission Spectroscopy (XPS) and X-ray diffraction (XRD). The chemical composition of the WO_3_ films was determined by XPS analysis. Figure 1a shows the W 4 *f* XPS results for the WO_3_ films grown on STO, which are fitted with just one doublet. The peaks at 35.8 and 37.9 eV are attributed to the W 4*f*_7/2_ and W 4*f*_5/2_ core-levels, respectively. The O1*s* spectrum can be fitted with a single peak at 530.5 eV as is shown in Fig. 1b. Both of these spectra are in agreement with reported values in the literature for stoichiometric WO_3_, indicating the complete oxidation of tungsten ions in the composition of the films^41^. The XRD patterns of a WO_3_ thin film and a blank STO substrate are compared in Fig. 1c. The peak at 22.8° is attributed to the (001) plane of the substrate and is seen in both patterns. The inset of Fig. 1c reveals that three peaks appear in the range of 23°–25°, indicating the presence of polycrystalline monoclinic WO_3_ (JCPS Card No. 075–2072). The peaks located at 23.1°, 23.7° and 24.2°are attributed to the (001), (020) and (200) planes respectively. These three peaks as well as their periodicity at higher angles reveals the high crystallinity of the film. The topography of the film determined by AFM microscopy shown in Fig. 1d reveals that the WO_3_ film was grown in Volmer-Weber mode with granular nature. With a thickness of 50 nm, the RMS roughness of the film is 0.5 nm, which is suitable for electronic applications in FET devices.

**Figure 2:**
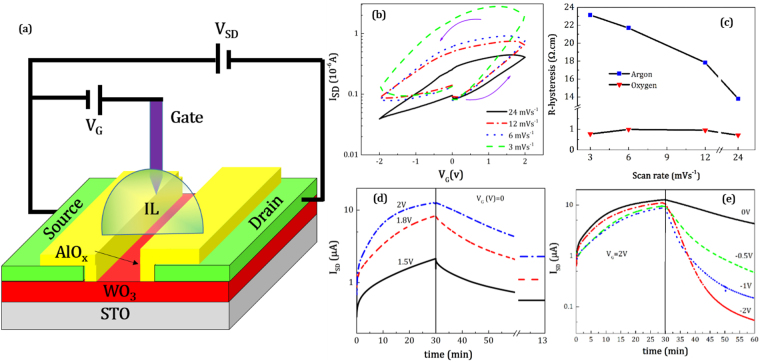
The measurements of *I*_SD_ from the IL assisted FET with WO_3_ film as electrical channel in argon gas. (**a**) A schematic of the field emission transistor device working with ionic liquid, (**b**) *I*_SD_ vs. gate voltage in different gate sweep rates in argon medium, (**c**) resistivity hysteresis (R-hysteresis) as a function of gate sweep rate in argon or oxygen, (**d**) *I*_SD_ current vs. time when a positive gate voltage is applied for 30 min with values varying from 1.5 to 2.0 V and then setting the gate voltage to zero. (**e**) *I*_SD_ vs. time after first applying a gate voltage of 2 V for 30 min and then applying a negative gate voltage varying from 0 to −2V.

IL gating of the WO_3_-based FET was first attempted in a glovebox filled with argon gas. The schematic of the FET device is shown in Fig. 2a. A low source-drain voltage of 0.1 V was used to ensure that the current is in its linear regime. A gate voltage in the range of ±2 V was applied between source and gate electrodes. The gate voltage was swept at four different rates of 3, 6, 12 and 24 mVs^−1^. The *I*_SD_ current passing from the WO_3_ channel is shown as a function of gate voltage in Fig. 2b and the channel resistivity switches between low and high values with positive and negative gate voltages. No threshold is observed in the measured range of values and the *I*_SD_ immediately increases when gate voltage is swept from zero to positive values for all sweep rates. Starting from a relatively low *I*_SD_ at *V*_G_ = 0 V (under 0.1 µA), it decreases by at least 1–2 orders of magnitude depending on the gate voltage sweep rate and reaches a metallic state at 2 V. This metallic state persists even when *V*_G_ is reset to zero. When V_G_ is swept to negative voltages, the *I*_SD_ returns to its initial value at *V*_G_ = 0 V. A clear hysteresis in the *I*_SD_ centered at *V*_G_ = 0 V is observed in all gate voltage sweeps. The hysteresis of the resistivity (R-hysteresis) here is defined as the difference between resistivity of the two states at *V*_G_ = 0 V (first state: the starting point, second state: when *V*_G_ returns to zero from positive values). The R-hysteresis as function of gate sweep voltage is shown in Fig. 2c. The remnant resistivity is high when the sweep rate is low and decreases from 23 to 13 Ω.cm with increasing rate. Surface electrochemical reactions are the likely source of carrier injection at the interface into the channel, and the penetration of dopants or the creation of oxygen vacancies which donate carriers is apparently increased with longer gating times.

The sweep rate dependence of I_SD_ with gate voltage reveals that a relatively long time is needed to move to a steady state when a gate voltage is applied. The time-dependence of the *I*_SD_ current with constant gate voltages in the range of 1.5–2 V is shown in Fig. 2d. Positive gate voltages were first applied for a duration of 30 min before being turned off and *I*_SD_ was recorded as a function of time. The results show that *I*_SD_ increases continuously, even after 30 min of applied positive voltage. When the gate voltage is switched off, *I*_SD_ decays exponentially with time for all gating conditions. This shows that the current is not stable after the channel is filled with carriers by applying a positive gate voltage. It takes a long time for the current to reach its steady state at *V*_G_ = 0 V and even after 2 hours the current is still decreasing. It was observed that the source-drain currents reach a stable value of 1 µA after 10 hours with no applied gate voltage. The gate current during the different gate sweeps is shown in Figure S2a. It is clear that the gate leakage current does not exceed the 6 × 10^−8^ A at all gate voltages which is much smaller than the *I*_SD_ current range. A similar response occurs at the negative gate voltages when first a positive voltage is applied and then a negative one. Figure 2e reveals that a negative gate voltage leads to a dramatic decrease in *I*_SD_. The *I*_SD_ equilibrium values at high voltage gating are less than those at low voltage gating, therefore, the final channel resistivity depends on the gate voltage. To determine the physical characteristics of the channel current for each gate voltage, the source-drain current is fitted with the following exponential function;

1 $${I}_{SD}={I}_{\infty }+({I}_{0}-{I}_{\infty }){e}^{-\frac{t}{\tau }}$$

where *τ* is the decay time, *I*_0_ and *I*_*∞*_ are the values of the source drain current at the initial and final times respectively. The fitted parameters are shown in Table 1. The resistivity of the film calculated from the final *I*_SD_ current is also presented in this table. The equilibrium resistivity of the resistivity at a gate voltage of −2 V is 57 Ω.cm. The I_on_/I_off_ ratio (where *I*_*on*_ = I_infinity_ (*V*_G_ = 2 V) and *I*_*off*_ = I_infinity_(V_G_ = −2V)) was determined to be ~300 under argon atmosphere.

**Table 1:** The results of fitting *I*_SD_ time dependence to Eq. 1.

*V*_G_ *(*V*)*	*I*_0(_μA)	τ(s)	I_∞_ μ(A)	Resistivity (Ω.cm)
**0**	13.106	1461	0.632	4.75
**−0.5**	13.101	324	0.581	5.16
**−1**	13.099	177	0.279	10.7
**−1.5**	13.098	158	0.128	23.4
**−2**	13.055	200	0.035	57

**Figure 3:**
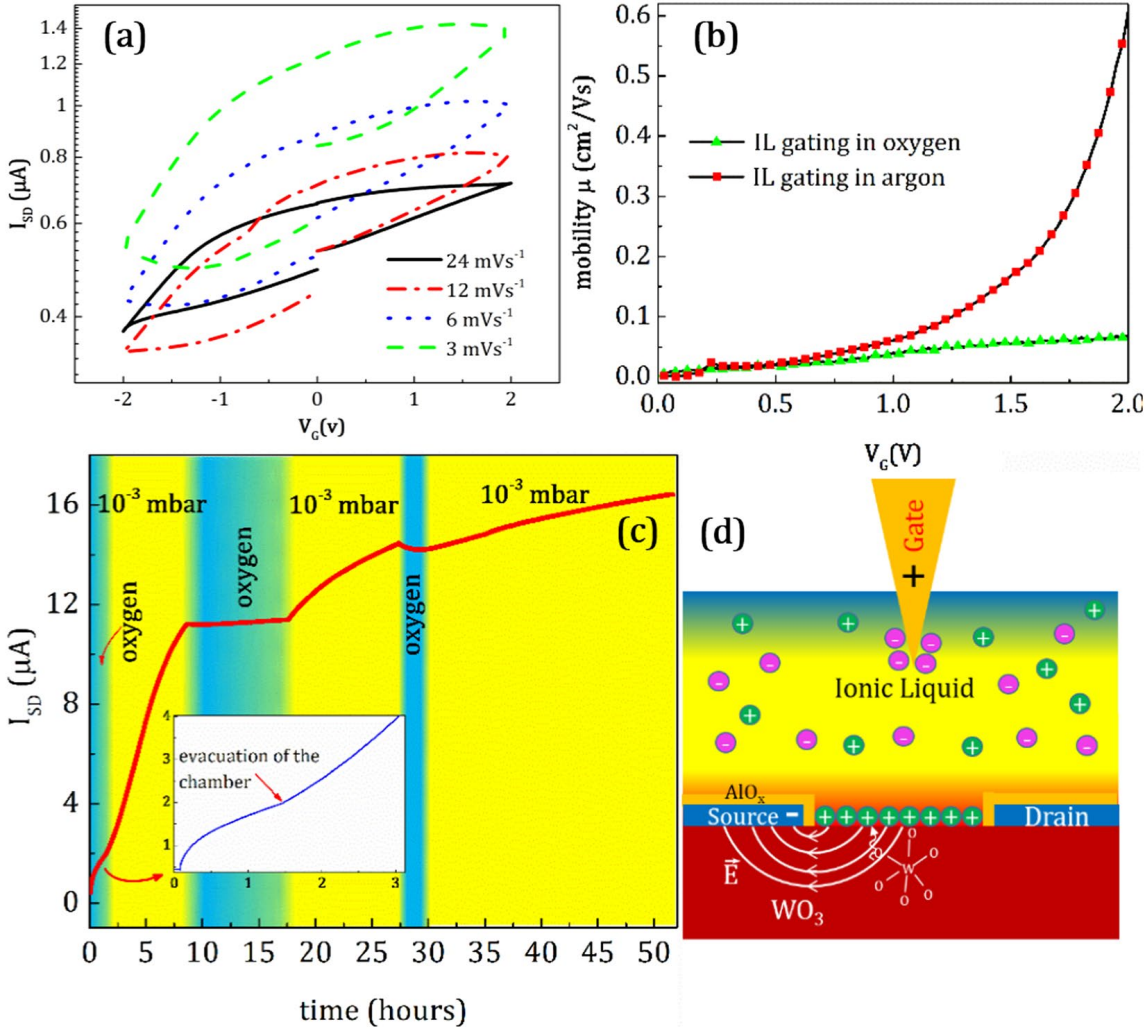
The measurements of *I*_SD_ from the IL assisted FET in oxygen gas and comparing the results with argon. (**a**) *I*_SD_ vs. gate voltage for different gate sweep rates in oxygen, (**b**) mobility in the linear regime calculated as a function of gate voltage in different gases, (**c**) source-drain current versus time when V_G_ was fixed at 2.0 V and the vacuum chamber of 10^−3^ mbar was filled with oxygen gas at a pressure of 200 mbar in different stages (blue colour shows the time when oxygen was introduced into the chamber), and (**d**) schematic of oxygen vacancy production in the WO_3_ film induced by the electric field.

The behavior of the WO_3_ channel in oxygen atmosphere is distinct from that in argon atmosphere. The current passing from the channel as a function of gate sweep voltage rate as measured in oxygen atmosphere is shown in Fig. 3a. The absolute deviations of *I*_SD_ are significantly smaller than the values measured in argon atmosphere. However, resistivity hysteresis is still seen at all scan rates. Figure 2c shows that R-hysteresis is essentially constant for all gate sweep rates, and it is ten times smaller in oxygen than it is in argon. The temporal *I*_SD_ current achieved by applying a 2 V gate voltage for 30 min and its behavior when negative voltages are applied is shown in Figure S3. Following from this, the *I*_*on*_/*I*_*off*_ ratio (*I*_*on*_ = *I*_SD_ (*V*_G_ = 2 V) and *I*_*off*_ = *I*_SD_ (*V*_G = _−2V)), was determined to be ~10 in oxygen which is 30 times smaller than in argon. The field-effect mobility including contributions from the film channel and the ionic liquid can be determined from our experimental results. The mobility in the linear regime which is defined at low source drain voltages (V_SD_ < 1 V) is given by the following equation^42,43^;

2 $${\mu }_{lin}=\frac{\partial {I}_{SD}}{\partial {V}_{G}}\frac{L}{W{C}_{IL}{V}_{SD}}$$

where *L* and *W* are length and width of the FET channel and C_IL_ is the capacitance per unit area of the IL which is determined to 7.0 μF·cm^−2^ for EMIM-TFSI^44^. The mobility from different gate voltage scan rates both in argon and oxygen calculated from the transconductance over the entire positive gate bias ranges are shown in Figure S4 and S5. In both curves it is apparent that the rate of increase of the mobility is higher at lower gate voltage scan rates. The gate voltage dependence of the mobility is relatively linear in oxygen and exponential in argon. The calculated mobilities with a gate scan rate of 3 mVs^−1^ in both argon and oxygen are compared in Fig. 3b. In argon atmosphere, applying a gate sweep rate of 3 mVs^−1^ leads to an increase in the carrier mobility from 0.003 cm^2^V^−1^s^−1^ in 0 V to 0.6 cm^2^V^−1^s^−1^ in +2 V. The increase in oxygen is much less pronounced, from around 0.04 cm^2^V^−1^s^−1^ at 0 V to 0.065 cm^2^V^−1^s^−1^ at +2 V, which is a change 10 times smaller than that in argon.

Figure 3c shows the direct dependence of *I*_SD_ on the oxygen pressure of the chamber during the IL gating of the FET device. The gate voltage was fixed at *V*_G_ = 2 V for the duration of the measurements and the chamber was first filled with oxygen to a pressure of 200 mbar. When the gate voltage is turned on, *I*_SD_ increases slowly and reaches a relatively stable current after 1.5 hours as can be seen in the inset of Fig. 3c. Then the chamber was evacuated to a pressure of 10^−3^ mbar. The *I*_SD_ current increases rapidly as the chamber reaches the base pressure. After 8.5 hours the chamber was again filled with oxygen to a pressure of 200 mbar, although the current was still rising. The current then remained relatively constant at 11 µA during the 8.5 hours when an oxygen pressure of 200 mbar was maintained in the chamber. When the chamber was pumped out again, the current increased in vacuum from 11 µA to 14.5 µA. At this point the current appears to have reached its maximum in vacuum. Upon filling the chamber with oxygen, the current decreased slightly. This experiment shows a clear correlation between the *I*_SD_ and the presence of oxygen in the chamber.

There are some issues that need to be addressed here. First of all, according to a model described by Nakano *et al*.^20^, the metallization of VO_2_ insulating films is attributed to the collective bulk carrier delocalization driven by electrostatic surface charge accumulation during the gating process. According to our results, the changes in current at a constant gate voltage for WO_3_ are very different. They are sluggish and hysteretic, meaning that the electrostatically induced charge carriers are injected slowly in the WO_3_ film. The high electric field produced at the interface of the WO_3_ film channel might be expected to create charge carriers much faster than we have observed, thus it is difficult to accept that the current changes are due to direct electrostatic charge injection. On the other hand, *I*_SD_ changes very little in an oxygen atmosphere but it changes significantly under in an argon atmosphere and in vacuum. Nakano’s^20^ model doesn’t explain the effect of oxygen on the gating of the WO_3_ film channel. In the dual-ion switch model proposed by Lu *et al*.^45^, the oxygen is inserted into the structure just in the negative voltage range. At positive voltages, hydrogen is responsible for lowering the resistance of the film. It is to be noted in our data that oxygen plays an important role to increase the conductivity of the film channel in the positive gate voltages. The results show that the metallization of WO_3_ decreases when the chamber and consequently ionic liquid are under oxygen. This indicates that the electrochemical reactions leading to the change in resistance are linked to the oxygen atoms, despite the proton intercalation proposed by Lu *et al*.^45^. Two types of oxygen atoms exist in the electric double layer of our FET device; one as a part of the anion in the formulation of the IL, and the other in the WO_3_ structure. Of these two, the oxygen in the WO_3_ structure is likely to be more sensitive to oxygen pressure. The oxygen atoms in the anion of EMIM-TFSI form double bonds with sulphur atoms, making them insensitive to the atmosphere during electrochemical reactions. When the gating experiment is done in oxygen, the IL is being saturated with oxygen atoms and the process of oxygen removal from the WO_3_ film to the IL is decelerated. The model of oxygen vacancies proposed by Parkin *et al*.^27^ describes our results on WO_3_ films quite well. In this model, the metallization of the channel is due to the penetration of oxygen vacancies or removal of oxygen atoms from the WO_3_ film due to the presence of the high electric fields at the oxide-ionic liquid interface. The oxygen vacancies lead to electronic doping. Figure 3d shows a schematic of vacancy production inside the WO_3_. The diffusion of oxygen vacancies increases with time when constant gate voltage is applied. As the channel undergoes an electrochemical reaction, the process of oxygen removal from the depth of the WO_3_ film takes time. The dynamic curves of *I*_SD_ in fixed gate voltages which were determined in argon showed that the higher positive gate voltages lead to a lower resistivity of the film channel. One can say that increasing the capacitance of ionic liquid may lead to a decrease in the electric field at the surface of the WO_3_ film. It should be noted that, this idea doesn’t contradict our hypothesis of the production of oxygen vacancies inside WO_3_ film structure, because decreasing the electric field can lead to the re-oxidation process inside the film. In the other words, decreasing the electric field at the interface of the ionic liquid and the WO_3_ film, leads to the decrease in *I*_SD_ current in oxygen atmosphere that is completely in agreement with our results.

**Figure 4:**
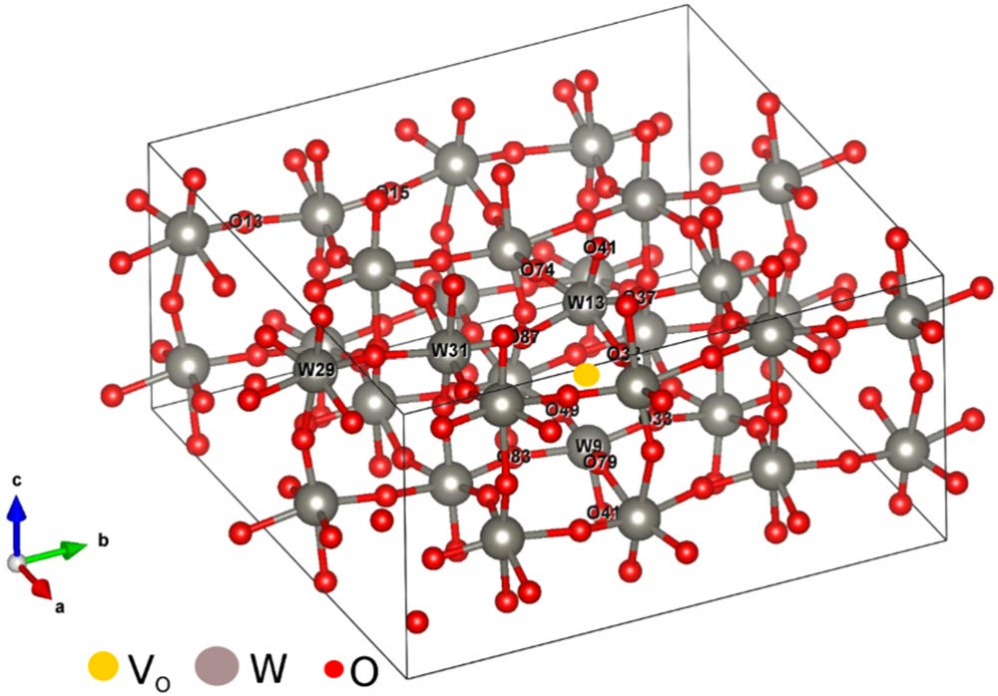
Periodically repeated supercell of WO_3_ including 3.12% oxygen vacancy. W13 and W9 lie in the vicinity of oxygen vacancy (VO), W31, W29, O13 and O15 are further away from Vo. The picture is prepared by VESTA^47^.

**Figure 5:**
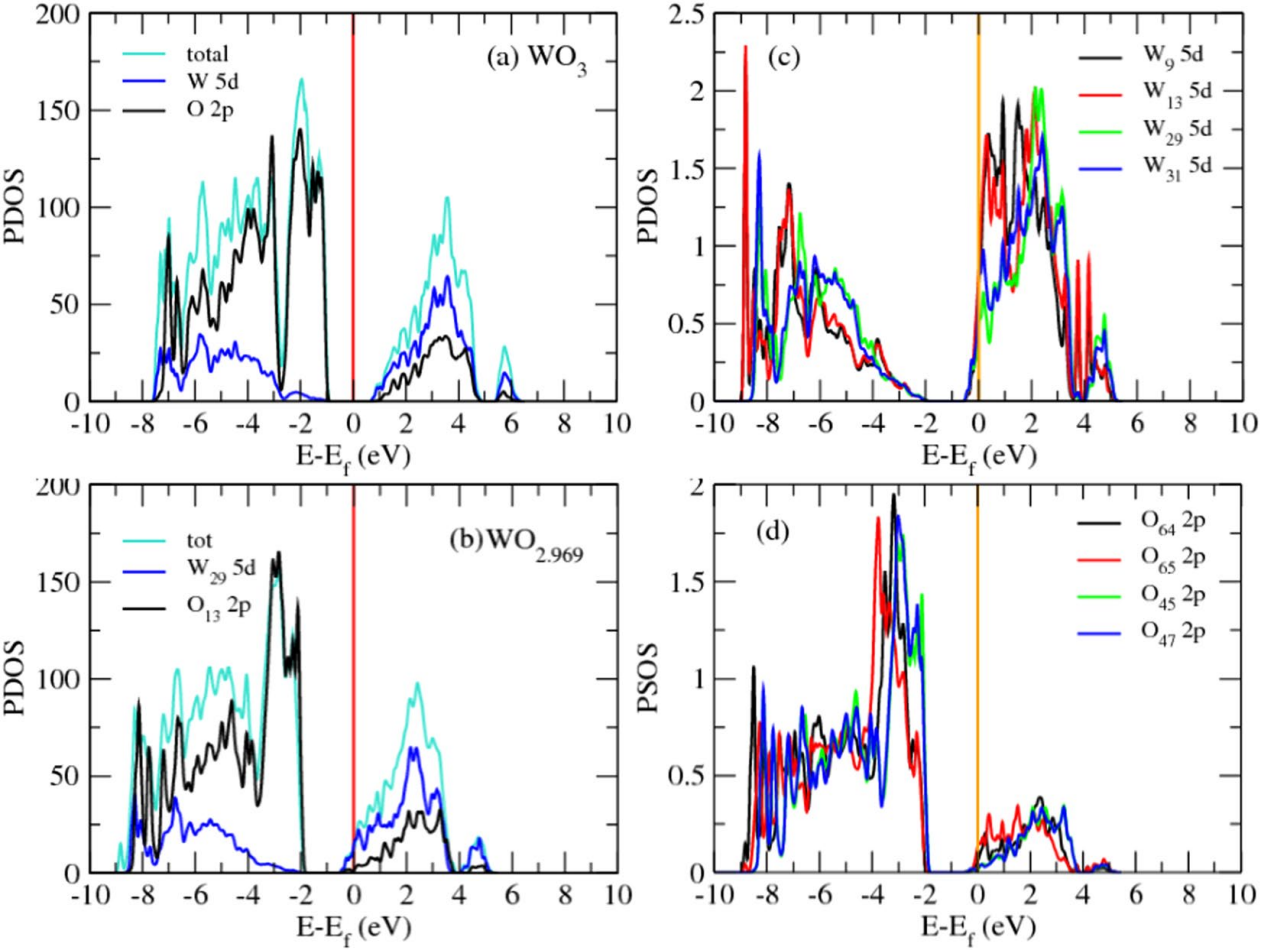
Calculated WO_3_ partial density of states for WO_3_ with different oxygen vacancies. (**a**) calculated WO_3_ partial density of states for (**a**) pure WO_3_, (**b**) WO_3_ with dilute oxygen vacancies, (**c**) W9 and W13 close to VO, W29 and W31 far from VO, and (**d**) O64 and O65 close to VO, O45 and O47 far from VO.

This metallization by oxygen vacancies was further examined via the Density Functional approach. The band structures of the stable phases of WO_3_ are similar^46^ but the room temperature monoclinic phase is more relevant than the others to this investigation. A 128 atom monoclinic supercell of WO_3_ with the vacancy concentration of 3.12% (as shown in Fig. 4) was studied by the density of states. The density of states calculations in Fig. 5 show that the valance band mainly consists of the 2*p* states of O mixed with 5d states of W. The O 2*p* states play the main role for the valence band nearest to Fermi energy. The contribution of the W 5*d* states to the conduction band minimum nearest to the Fermi energy is greater than that of O 2*p* states. There are six inequivalent oxygen sites in the unit cell. Introducing an oxygen vacancy to the structure causes the Fermi level to be shifted up into the conduction band minimum (CBM) depending on the number of valance bands. Six electrons should leave the system upon the removal of one oxygen. We should therefore lose 3 states in our system. It seems however that we have lost more than three, causing the Fermi level to shift to around 0.78 eV higher than CBM^46^. In a pristine system, the band gap in our calculation is around 1.03 eV while in a system with 3.12 percent oxygen vacancies the gap decreases to 0.79 eV. This oxygen vacancy mediated shrinkage in the bandgap has been verified experimentally^48,49^. The O64, O65, W9 and W13 lie near the oxygen vacancy (Vo) while O45, O47, W29 and W31 lie far from Vo.

Calculated W and O partial DOS shows that near and far tungsten atoms play almost same role in conductivity, both having similar partial DOS at the Fermi energy, while near and far oxygen atoms do not play the same role in conductivity because their partial DOS at the Fermi energy is not the same (Fig. 5c). The role of near oxygen atoms in conductivity is higher than far oxygen atoms.

**Figure 6:**
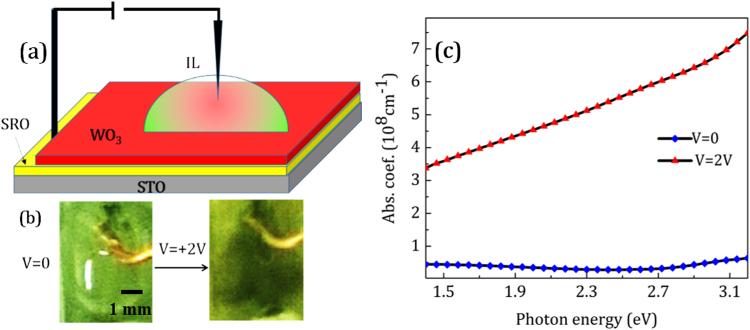
Change in the colour of WO_3_ surface when a DC potential is exerted to the IL at the fabricated device. (**a**) Schematic of the device fabricated to trace the WO_3_ surface colouration effects, (**b**) the image of the WO_3_ surface in contact with the ionic liquid when different voltages are applied, (**c**) Absorption spectra of the WO_3_ surface when different voltages are applied at visible wavelengths.

The metallic behavior of WO_3_ films mediated by oxygen vacancies should cause some changes in the color of the film as in the in electrochromic effect^34,50,51^. To see the changes in the coloration of the films, WO_3_ was deposited on SrRuO_3_ (SRO) conductive thin layers (the deposition method is described in the supplementary information). The schematic in Fig. 6a shows the structure used. When a DC electric voltage of magnitude 2 V is applied between the ionic liquid and the SRO for just 5 minutes, the color of a part of WO_3_ film in contact with IL switched from its colorless state to a dark blueish color. Figure 6b shows the change in the coloration of the WO_3_ surface by applied DC voltage. This color change was reversed by turning the voltage off or applying a negative voltage to the device. Figure 6c shows the absorption coefficient determined by ellipsometric spectroscopy in the visible range. It is shown that the absorption coefficient increases under applied voltage which demonstrates that the color of the surface undergoes a pronounced change. Wu *et al*.^32^ and ViolBarbosa *et al*.^34^ have reported that the conducting WO_3_ film produced by biasing using an ionic liquid is colourless. The color of the surface didn’t change when oxygen vacancies are induced in the WO_3_ structure, which seems to contradict our findings. However the conductivity of our ungated films is greater than that reported in those references, likely reflecting a greater initial vacancy concentration. It should be noted that there is much experimental evidence for the relation between oxygen vacancies and the chromic properties of WO_3_ films^34,46^. It can be said that WO_3−x_ films exhibit different chromic properties for different levels of oxygen deficiency. For example, if x > 0.5, films are metallic and conductive. If 0.3 < x < 0.5, films are blue and conductive, and finally, if x < 0.3 films are transparent and resistive^52^. It is likely that the level of oxygen vacancy production is different for the different films, depending on their structure, roughness and morphology.

## Experimental Presedures

### Film Preparation

WO_3_ films were grown by PLD on (001)-oriented 10 × 10 mm^2^ SrTiO_3_ substrates supplied by Crystal GmBH. The optimal conditions to obtain the smooth films were discussed in our previous report^40^. High-purity WO_3_ powders (Sigma-Aldrich, >99.99%) were pressed (150 bar) and sintered (900 °C) for 8 hours to form a target of diameter 20 mm. In order to deposit thin films, the PLD chamber was first evacuated to a base vacuum of 10^−6^ mbar before introducing pure O_2_ gas (99.99%) at a pressure of 60 µbar. A KrF excimer laser with a pulse duration of 23 ns and a wavelength of 248 nm was used. The repetition rate and the laser energy fluence were 3 Hz and 0.6 J/cm^2^ respectively. The WO_3_ films were deposited at a substrate temperature of 600 °C. For each deposition, 3000 laser shots were delivered to the target and a 50 nm thick film was obtained.

### Liquid Gating Experiments

The ionic liquid used in this study was 1-Ethyl-3-methylimidazolium bis (trifluoromethylsulfonyl) imide (EMIM-TFSI) which was purchased from Sigma Aldrich. Before using the IL, it was baked at 100 °C in a vacuum chamber at a pressure of 10^−6^ torr for several days in order to remove absorbed water molecules. The IL was then immediately transferred to a glovebox which is under a controlled argon atmosphere, and it was stored there. All of the experiments in this study were carried out under dry atmospheres of either argon (in the glovebox), oxygen or in vacuum.

### Device fabrication

Source and drain electrodes were deposited on the surface of WO_3_ films by DC magnetron sputtering in a Shamrock multi-chamber deposition system. 4 nm Ti and 6 nm Pt films were deposited separately using a Kapton tape mask to create a channel with a size of 1 mm × 5 mm. An insulating AlO_x_ layer with a thickness of 50 nm was then deposited by sputtering in order to prevent any contact between the IL and the electrodes during the experiment. An optical image of the film after deposition is shown in Figure S1. A top-gate electrode consisting of a tungsten tip which was brought into contact with ionic liquid was used during the measurements. The resistivity of the transistor channel was measured using a constant source-drain voltage of 0.1 V.

### Characterization and measurements

X-ray photoelectron spectroscopy (XPS) measurements were carried out in ultra-high vacuum, using Al K_α_ X-rays from a twin anode source and a VG Scientific CLAM2 energy analyser. The topography of the surface was examined using contact mode atomic force microscopy (AFM). The X-ray diffraction (XRD) analysis was carried out using a Panalytical X’Pert Pro Cu Kα radiation source (λCu = 0.15405 nm) in the θ–2θ geometry. The current and voltage measurements were determined using Keithley 2400 sourcemeters during the IL gating experiments. A SOPRA GESP5 ellipsometer was used to measure the differential changes in amplitude ψ and phase Δ of incident polarised light as a function of incident angle from 40° to 65°. The absorption coefficient spectra were then calculated from those results in the energy range of visible light (1.4–3.2 eV).

### Theoretical Calculations

All calculations presented here to investigate the impact of oxygen vacancies were carried out using plane-wave based Density Functional Theory (DFT) as implemented in the Quantum Espresso^53^ package. The kinetic energy cutoff for wave functions was set to 40 Ry. The projector augmented wave (PAW) pseudo-potentials were employed with the following configuration: 4*f* ^14^ 5*d* ^4^ 6 *s* ^2^ for W and 2 *s* ^2^ 2*p* ^4^ for O. All pseudo-potentials were generated from PS library 0.3.1 employing the Perdew-Burke-Ernzerhof^54^ (PBE) exchange-correlation. A 2 × 2 × 4 Gamma centered k-point mesh in the Brillouin zone was sampled in the calculation. A 128 atom monoclinic supercell of WO_3_ (32 tungsten atoms, 95 oxygen, 1 oxygen vacancy) corresponding to a nominal vacancy concentration of 3.12% was studied by the density of states and partial density of states (see Fig. 4). Drawings in Fig. 4 were produced by VESTA^47^.

## Conclusions

A FET device structure was fabricated from WO_3_ films deposited on SrTiO_3_ substrates utilizing ionic liquid gating. Gate voltage in an argon atmosphere decreases the resistivity of the channel by more than two orders of magnitude. When the experiment is performed in an oxygen atmosphere, the decrease in the resistivity is much less. Metallization is attributed to the removal of oxygen atoms from the WO_3_ surface under applied electric field, and donation of the two electrons from the neutral vacancy to the 5*d*(W) conduction band. DFT calculations confirm that the electronic DOS is strongly affected by oxygen defects in the WO_3_ lattice and the lowest conduction bands are dominated by hybridized W 5*d* and O 2*p* states which govern the DOS at the Fermi energy. The model of oxygen vacancy formation as a cause of metallization of the surface of WO_3_ is supported by the electrochromic behavior of the films. This effect confirms that WO_3_ thin films are potential candidates for electrochromic applications.

## Electronic supplementary material

Supplementary
