# Supplementary material for: Oxygen Vacancy in WO3 Film-based FET with Ionic Liquid Gating
Source: Sci Rep. 2017 Sep 25;7:12253. doi: 10.1038/s41598-017-12516-y (PMC5612984; doi:10.1038/s41598-017-12516-y)
Supplement: Supplementary file 1 — Supplementary [file 41598_2017_12516_MOESM1_ESM.pdf]

Supplementary Information for

Oxygen Vacancy in WO_3 Film-based FET with Ionic Liquid Gating

Hossein Kalhori[a,b], Michael Coey[a], Ismaeil Abdolhosseini Sarsari[b,c], Kiril Borisov[a], Stephen Barry Porter[a], Gwenael Atcheson[a], Mehdi Ranjbar[b], Hadi Salamati[b], Plamen Stamenov[a]

[a] School of Physics and CRANN, Trinity College, Dublin 2, Ireland.

[b] Department of Physics, Isfahan University of Technology, Isfahan, 84156-83111, Iran.

[c]Computational Physical Sciences Research Laboratory, School of Nano-Science, Institute for Research in Fundamental Sciences (IPM), P.O. Box 19395-5531, Tehran, Iran.

Deposition of $SrRuO_3$ films: $SrRuO_3$ (SRO) films were grown by PLD method on (001)-oriented 10×10 mm^2 STO substrates supplied by Crystal GmBH. High-purity SRO powders (Sigma-Aldrich, >99.99%) were pressed (150 bar) and sintered (1100˚C) for 12 hours to form a target of diameter 20 mm. In order to deposit thin films, PLD chamber was first evacuated to a base vacuum of 10^{-6} mbar, and then pure O_2 gas (99.99%) of 0.1 mbar was introduced to it. A KrF excimer laser with a pulse duration of 23 ns and a wavelength of 248 nm was used. The repetition rate and the laser energy fluence were 5 Hz and 1.4 J/cm^2 respectively. The SRO films were deposited at substrate temperature of 700 ˚C. The films with thicknesses of 10 nm was obtained by this method.

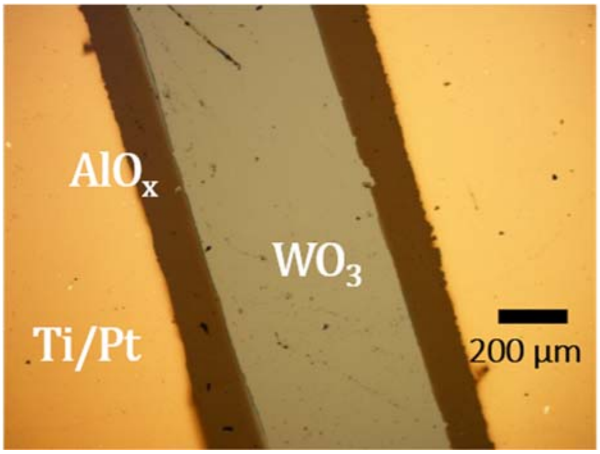

Fig. S1 Optical microscope image of the surface of transistor channel

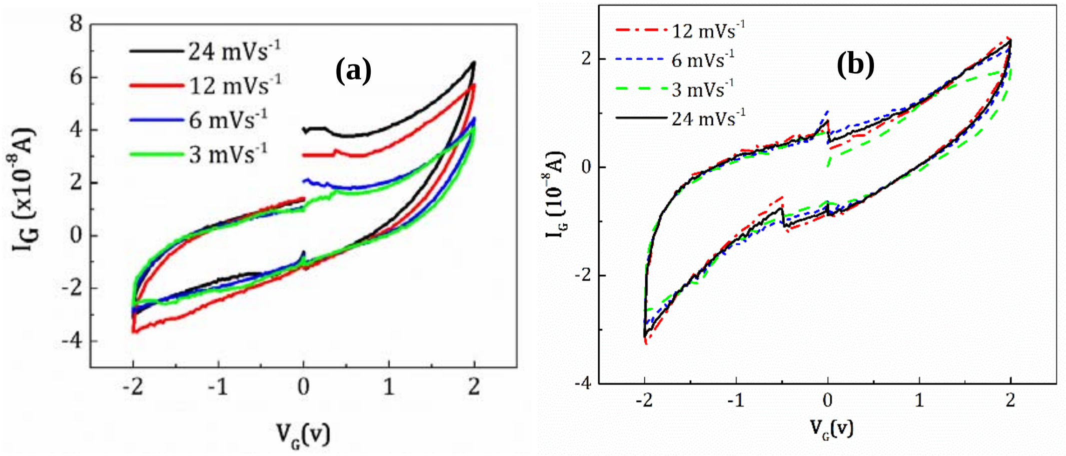

Fig. S2 Gate current (I_G) as a function of gate voltage with different sweep rates in (a) argon and (b) oxygen atmospheres.

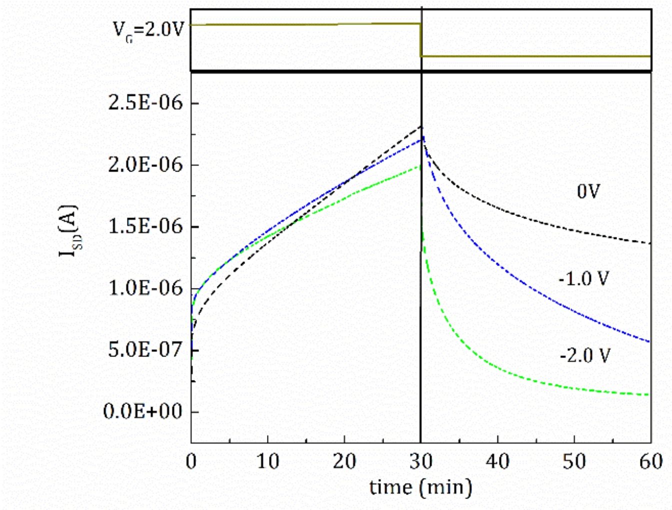

Fig. S3 Temporal changes of I_{SD} vs time in oxygen after a gate potential of 2 V is applied for 30 min and it turns to a zero or negative voltage. Top panel shows the gate voltage change vs. time,

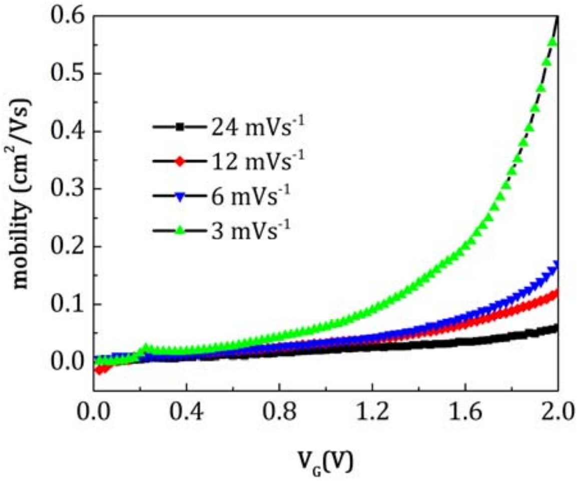

Fig. S4 Mobility in the linear regime as a function of gate voltage in different sweep rates in argon.

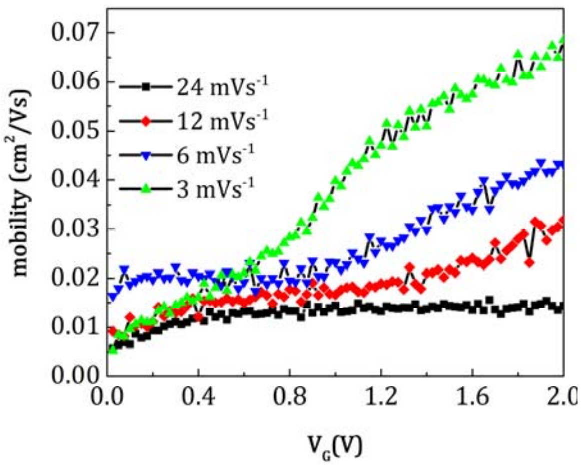

Fig. S5 Mobility in the linear regime as a function of gate voltage in different sweep rates in oxygen.
